# Story of Lyari

**DOI:** 10.4269/ajtmh.19-0839

**Published:** 2020-04

**Authors:** Rabab Batool

**Affiliations:** Paediatric Research and Child Health, Aga Khan University, Karachi, Pakistan

On Monday May 6, 2019 at 11:30 am, I was standing in the center of Khadda Memon Market, the oldest known market in the center of Lyari Town. Lyari is a densely populated urban slum settlement, where most cases of extremely drug-resistant (XDR) typhoid were reported. I had to make an announcement, broadcast through a megaphone, about the ongoing typhoid vaccination campaign in Lyari. I was reluctant and cautious as I noticed the diverse ethnicities of people shopping.

I was standing on this corner in Lyari Town because my hospital, Aga Khan University Hospital in Karachi, had decided to conduct a massive vaccination campaign to fight the XDR typhoid outbreak that started in Hyderabad in 2016 and rapidly spread to neighboring Karachi, including Lyari Town. After two full years of people dying, the outbreak was still not contained. And so, I stood looking out at Lyari Town. Lyari has diverse ethnic groups because it is a slum area. Looking out, I realized the town is mostly populated with Afro-Indians. The Sheedis are believed to be descendants of slaves from Africa who arrived in Pakistan between 1200 and 1900 with Arab invaders. They are dark skinned, curly haired, huge, speak a slang called street Urdu, and are mostly fishermen and laborers. The blue-collar workers at the market reflected the spectacular ethnic, linguistic, religious, and sectarian diversity such as Baloch, Sindhis, Lasis, Katchis, and Memons. These people are now locals in Lyari, but they or their families come from different areas of Pakistan. For example, the Baloch are from Balochistan Province; Sindhis, Lasis, and Katchis are from Sind Province; and Memons migrated to Pakistan from India at the time of partition. Because the cost of living is less in slums like Lyari than in other parts of the city, less privileged people travel from small cities to big cities like Karachi, settle in these slums, and search for work.

Looking around, I see small heaps of rubbish everywhere. A few meters away, an open sewage hole was overflowing. I had never been to Lyari before. I am a Punjabi (from Punjab Province). I have lived in Sind all through my life. My sister is married to a Sindhi. My cousin is married to a Balochi, and my best friend is a Memon. Although I can only speak the national language, we all share the same nationality. I am one of them.

A team member told me there were warehouses and industrial units storing and manufacturing perilous materials such as chemicals, industrial bleach, detergents, acid, and plastic that have intensified the agonies of the people living in this overcrowded locality. That day, I understood why the highest number of XDR typhoid cases was from Lyari.

Because it was May and schools were closed for summer vacation, we were doing everything to engage the community and increase vaccination uptake through the health facilities. I was holding a blue megaphone in my hand. I put the megaphone to my lips, gathered all my courage, and yelled, “Your town has been struck by an outbreak of extensively drug resistant typhoid. It’s a lethal disease that is difficult to treat and may cause serious complications. We have started a vaccination campaign at Lyari General Hospital, where your children can receive free of cost vaccination for typhoid fever. Please bring your children, Monday to Saturday from 9 am till 2 pm.”

My team members were distributing handbills with messages about the vaccination campaign. Over the past month, we had already arranged community-level meetings, Whatsapp and Facebook groups, school-based vaccinations, and informational lectures. With the high rates of absenteeism in the schools and now summer vacation, we had to leave the schools and head out to the street corners and other places in the community. Later, we started highly effective mobile vaccination camps. The mobile vaccination camps increased the uptake and helped us reach the underserved populations. The camps also allowed us to directly interact with the parents, address their questions, and win their trust.

Standing on the corner, I wondered how many people understood me. I started distributing the handbills with my team. I was trying to talk to women to find out where they lived and if they wanted to vaccinate their children against typhoid. If not, why? I also wanted to know where they got their drinking water from and how they treated it?

While I was telling people about how typhoid spreads and ways to prevent it, a teenager came to me and stood nearby. He was listening carefully. When I noticed him, I smiled. He came closer and started talking to me. He was a 15-year-old, tall, Sheedi boy with a pleasant personality, curly hair, and beautiful brown skin. He said, “Madam, you guys are doing a great job. I had typhoid. Do you want to know what it did to me?” I looked at him clueless. He raised his shirt from his stomach and showed me a big scar from surgery. He said, “I am the only son of my mother. My father was killed in a Lyari gang war when I was 5 years old. The only legacy my father left for my mother was a one-bedroom house that she had to sell to get me treated at a private hospital.” He looked very sad. He said, “Can I have some handbills? I will distribute them to my neighbors, friends, and relatives.” No one should have to suffer the effects of this devastating disease.

Since 2016, thousands of children suffered from this drug-defying strain of typhoid in Pakistan. Many families had to suffer financial hardships to bear the cost of the treatment for their children and out-of-pocket expenses.

The effectiveness of the vaccination campaign to control the outbreak is undeniable. Eighty-seven thousand five hundred sixty-eight children in Lyari Town were vaccinated with typhoid conjugate vaccine (TCV)-Typbar. The coverage of TCV-Typbar in Lyari Town was found to be 80% in a post-campaign vaccination coverage survey. The surveillance data show that the incidence of typhoid fever has declined significantly among children after the immunization campaign in Lyari Town.

The current Typhoid outbreak indicates the failed water and sanitation system in Pakistan. A multipronged approach comprised of vaccination of children and adolescents, water, sanitation, and hygiene interventions along with health education and capacity building for diagnosis and treatment needs to be implemented at the national level. Municipal and provincial governments need to take initiative to improve water and sewage infrastructure in the country.

